# Role of Natural Antioxidant Products in Colorectal Cancer Disease: A Focus on a Natural Compound Derived from *Prunus spinosa*, Trigno Ecotype

**DOI:** 10.3390/cells10123326

**Published:** 2021-11-26

**Authors:** Maria Condello, Stefania Meschini

**Affiliations:** National Center for Drug Research and Evaluation, National Institute of Health, 00161 Rome, Italy

**Keywords:** colorectal cancer cells, antioxidant products, natural compounds, *Prunus spinosa*

## Abstract

Colorectal cancer (CRC) is on the rise in industrialized countries, which is why it is important to find new compounds that are effective, with little or no adverse health effects. CRC arises from some cells of the epithelium which, following a series of genetic or epigenetic mutations, obtain a selective advantage. This work consists of a review on endogenous and exogenous antioxidant products that may have an efficacy in the treatment of CRC and an experimental study, in which the treatment was carried out with a natural compound with antitumor and antiproliferative activity, *Prunus spinosa* Trigno ecotype, patented by us, on HCT116 colorectal carcinoma cell line. The superoxide content was quantified after the treatments at different concentrations (2, 5, or 10 mg/mL) by means of the DHR123 probe; loss of the mitochondrial membrane potential with the tetramethylrodamine methyl ester (TMRM) cationic probe and reduced glutathione content (GSH) from monochlorobimane (MCB). This study revealed the importance of a careful choice of the concentration of the natural compound to be used in the CRC, due to the presence of a paradoxical effect, both antioxidant and pro-oxidant, depending on the different physiological conditions of the cell.

## 1. Introduction

Colorectal (CRC) is considered the third cancer in terms of recognition and the second in terms of mortality in the world. The level of incidence is strongly correlated with the socio-economic development of the country and the lifestyle of the population. It is difficult to understand how the incidence of this cancer is higher in more developed countries due to demographic aging and an unhealthy diet. The main causes of incorrect behaviors that favor the onset of CRC are: diet low in fiber, fruit and vegetables, calcium and vitamin D; overweight and obesity; physical inactivity; alcohol consumption; alteration of the intestinal microbiota; age; gender and race [1].

CRC carcinogenesis also involves genetic alterations, but only 20-30% of patients have a family history. Often in many patients, a benign neoplastic lesion is initially observed, such as an adenomatous or serrated polyp which will then evolve as a result of mutations in the tumor. Numerous studies have shown that CRC result from malignant transformations of intestinal stem cells or intestinal cells that assume the characteristics of stem cells after malignant transformation [2].

Cancer cells are well-known for having a high level of reactive basal oxygen species (ROS), which is why multiple ROS signaling pathways are activated, resulting from metabolic events occurring at the level of intracytoplasmic organelles such as mitochondria, peroxisomes and endoplasmic reticulum. In addition to activating signaling pathways other than normal cells, cancer cells have a higher level of antioxidant enzyme systems to eliminate free radicals produced during rapid cell proliferation [3]. In healthy cells, mitochondria generate radical species that are detoxified by endogenous antioxidants, helping to keep their levels low.

Exogenous ROS, unlike endogenous ones, derive from the exposure of cells to toxins, stress, smoking and incorrect lifestyles that can lead to tumor transformation through the induction of DNA mutations, protein alteration and lipid peroxidation. Unfortunately, if the antioxidant defenses fail to counteract these effects, ROS lead to the transformation of the normal cell into a tumor [4].

Highlighting the cellular mechanisms involved in the induction of oxidative stress and understanding how it is possible to combat harmful metabolic events to prevent and avoid pathological conditions is the first objective of this review. 

The second goal is to emphasize the importance of endogenous enzymatic and non-enzymatic antioxidants and exogenous antioxidants in the onset of CRC.

As stated earlier, a diet lacking in fruits and vegetables has been associated with the onset of colon cancer. An important class of compounds found in these foods are exogenous antioxidants called flavonoids, the consumption of which has often been associated with reducing the risk of CRC [5].

Finally, we present an experimental analysis performed in our laboratory, using colorectal carcinoma cell line (HCT116), treated with a natural compound derived from *Prunus spinosa*, Trigno ecotype (Trigno M) [6].

These experimental data definitively confirm the role of flavonoids of the active component of Trigno M on oxidative stress, in the form of a pro-oxidant or antioxidant reaction, after in vitro treatment at the different concentrations used.

## 2. Endogenous Antioxidants 

Antioxidants are found inside our body (endogenous) or they can be introduced from the outside into the human body (exogenous). Regardless of the source, they have the unique ability to reduce the effects induced by ROS.

**Figure 1:**
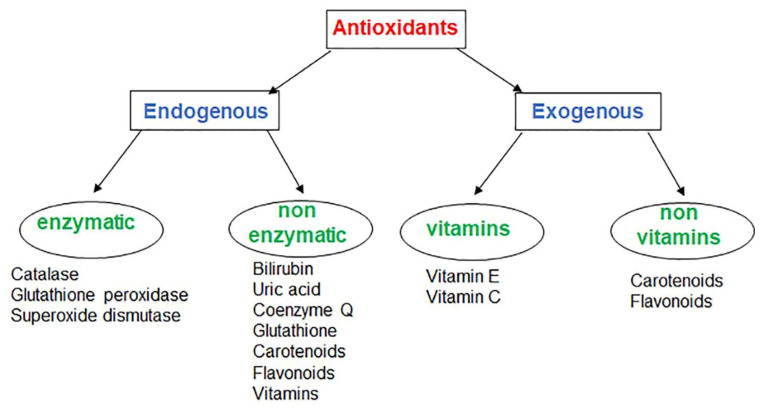
Classification of antioxidants.

Endogenous antioxidants have enzymatic (such as catalase, glutathione peroxidase, superoxide dismutase) or not enzymatic (bilirubin, uric acid, coenzyme Q, GSH, carotenoids, flavonoids, vitamins) activity that act on different cellular targets (Figure 1).

### 2.1. Endogenous Enzymatic Antioxidants

**Figure 2:**
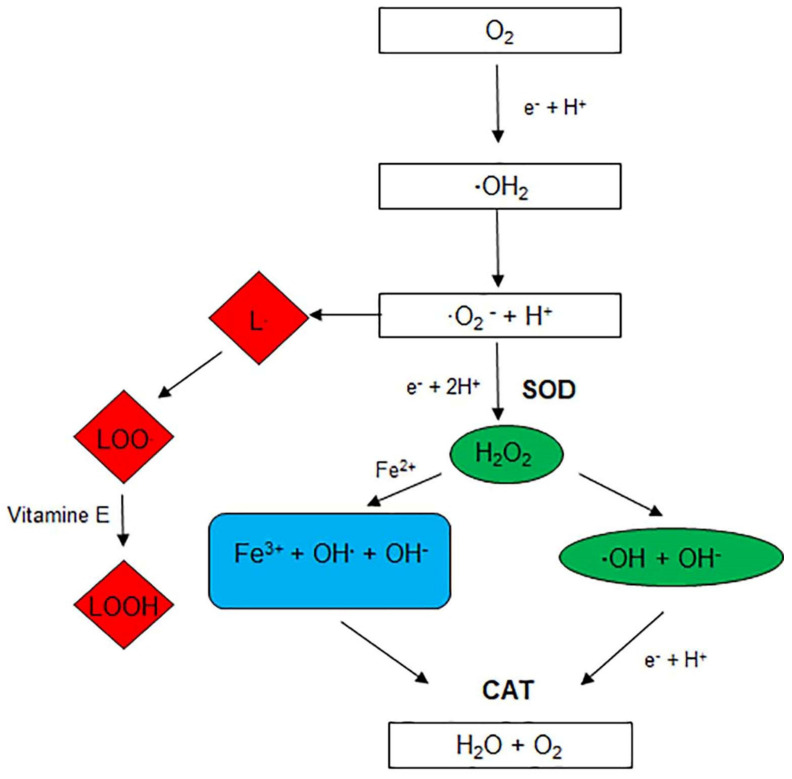
Schematic representation of the main reactions in which antioxidants are involved. Lipid peroxidation (red); Haber-Weiss reaction (green); Fenton reaction (light blue); SOD: superoxide dismutase; CAT: catalase.

The main reactions involving antioxidants are illustrated in Figure 2.

In particular, catalase (CAT), an enzyme located in the peroxisomes, converts hydrogen peroxide into water and molecular oxygen using iron or manganese as cofactors. Although the role of catalase in tumor is contradictory, some authors reported an increase of catalase levels in colorectal cancer [7]. 

Glutathione peroxidase (GPx), located in cellular mitochondria, converts hydrogen peroxide into water and fatty acid peroxides to alcohols using glutathione (GSH) as substrate.

ROOH+2GSH→ROH+GSSG+H_2_O (1)

Since GPx deficiency leads to excessive oxidative stress resulting in damage to proteins and lipids, it has been attributed a prevention role in cancer progression [8].

Superoxide dismutase (SOD), located in the cytosol and mitochondria, catalyses the dismutation of two molecules of superoxide radicals (O_2_^−^) to hydrogen peroxide and oxygen molecule (O_2_).

2O_2_^−^+2H^+^→2H_2_O+O_2_ (2)

Cytosolic SOD uses zinc and copper as cofactor, while mitochondrial SOD uses manganese. Many studies showed changes of SOD activity related to gradual progression of CRC; in particular SOD levels proportionally increased with the increase of grade of differentiation of CRC [9].

### 2.2. Endogenous Non-Enzymatic Antioxidants 

A second antioxidant line of defense, such as Glutathione, scavenges active free radicals by donating electrons and inhibiting the propagation of oxidative stress. Glutathione is a tripeptide of gamma-glutamyl-cysteinyl-glycine, the major cellular thiol protein and is found in all cell compartments. It has two forms: the reduced form (GSH) and the oxidized form (GSSG).

Under physiological conditions, reduced GSH is the predominant form, with a concentration up to 100-times higher than the GSSG form. GSH is synthesized in the cytosol by GSH synthetase (GSS) and then distributed to different organelles, where it performs a specific function.

**Figure 3:**
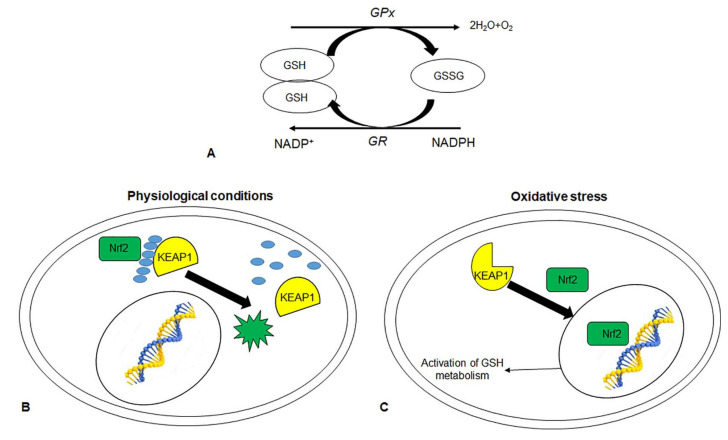
Schematic representation of Glutathione. (**A**) interconversion between the reduced (GSH) and oxidized (GSSG) form of Glutathione. The role of nuclear factor-2 related erythroid factor-2 (Nrf2) in GSH metabolism under physiological conditions (**B**) and oxidative stress (**C**). GSH: reduced glutathione; GSSG: oxidized glutathione; GPx: glutathione peroxidase; GR: Glutathione reductase; KEAP1: Kelch-Like ECH Associated Protein 1.

Under oxidative stress, GSH is converted by GSH-dependent peroxidases, such as glutathione peroxidase (GPx) and glutathione-s-transferase (GST), into GSSG. Glutathione reductase (GR) restores cellular GSH by converting GSSG to GSH at the expense of NADPH (Figure 3A). The metabolism of GSH removes and detoxifies the cells by carcinogens, so altering the pathway has an effect on cell survival. In particular, the excess level of GSH promotes tumor progression and elevated levels are associated with an increase in metastasis.

GSH and associated enzymes are controlled by a transcription factor, nuclear factor-2 related erythroid factor-2 (Nrf2). Under physiological conditions, Nrf2 binds Kelch-Like ECH Associated Protein 1 (KEAP1), via a Cullin3 adapter protein, leading to the ubiquitination and degradation of Nrf2 (Figure 3B). Under oxidative stress, the altered conformation of KEAP1 disrupts its association with Nrf2, with subsequent stabilization of Nrf2. Free Nrf2 translocated to the nucleus where it activated antioxidant response genes responsible for the synthesis and metabolism of GSH, antioxidant proteins such as GPX, drug-metabolism enzymes (Figure 3C) [10]. Not surprisingly, mutations in Nrf2 and Keap1 genes are responsible for altered expressions of antioxidant genes that promote cancer progression, as for CRC [11].

Furthermore, GST, which binds both GSH and drugs, is involved in cancer multidrug resistance. GSH-GST-drug conjugated is associated with multiple resistance-associated protein transporters (MRP1), which release drugs out of the cells [12]. For example, elevated levels of MRP in conjunction with GST were expressed in the oxaliplatin-resistant human colon cancer line compared to the parental cell line [13].

Another example of non-enzymatic exogenous antioxidants is unconjugated bilirubin, the end product of the Heme catabolic pathway. Heme, the precursor of hemoglobin, produced by the action of the alanine synthase, is converted into biliverdin by heme oxygenase enzymes. Biliverdin is then converted to unconjugated bilirubin by biliverdin reductase. Although increased levels of bilirubin have always been known as a sign of liver disorders, most recently bilirubin, along with uric acid, is considered the most potent endogenous antioxidant due to its continuous recovery in the bilirubin/biliverdin redox cycle [14]. Recent data strongly indicate that mean bilirubin levels may be protective against diseases associated with increased oxidative stress, such as CRC [15]. Although in vitro and in vivo works recognizing the importance of antioxidant and anti-inflammatory properties of bilirubin, epidemiological studies need further evidence to confirm or refute potential associations between circulating serum bilirubin level and CRC risk [16,17,18,19].

Uric acid is a product of purine metabolism [20]. Serum uric acid levels, measured in preoperative phase, were statistically higher in CRC patients, so uric acid may be a prognostic factor for CRC [21].

Other endogenous antioxidants with non-enzymatic activity, such as carotenoids, flavonoids and vitamins will be discussed in the exogenous antioxidants section.

### 2.3. Endogenous Antioxidants Related to CRC Prevention and Treatment

Considering the important role of endogenous antioxidants in the processes of cancerogenesis and metastasis, many studies have highlighted the importance of these compounds as potential targets for the prevention of primary and secondary cancer.

A recent study showed the beneficial effect of physical activity on antioxidant cell systems that counteract cancer-related aberrant gene expression [22]. The authors highlighted the beneficial effect of physical activity on the expression of cancer-related genes by improving the redox cell system. Metastasis-associated lung adenocarcinoma transcript 1 (MALAT1) is a long and highly expressed non-coding RNA sequence in many tumors, such as colorectal cancer. Its expression is increased in tumor metastases and is associated with reduced patient survival, indicating its potential prognostic value [23]. The study documented, for the first time, the downregulation of MALAT1 in healthy human subject after physical exercise and a significant negative correlation between MALAT1 and SOD expression on lymphoblastic cells [22].

Since oxidative stress is the cause of many tumors, redox biomarkers are very important in clinical practice. In a recent work, SOD, CAT, GPx, GR, uric acid, GSH, and oxidative damage products were measured in serum/plasma samples from CRC patients. The analysis showed that SOD activity and uric acid levels were higher in CRC patients than in healthy controls, suggesting an adaptive response to increased ROS. Whereas the activity of CAT, GR, GPx, and GSH was considerably lower because free radicals can inactivate and decrease their activity [24]. The possibility of being able to use these endogenous antioxidants as potential biomarkers could be useful in pre-operative diagnosis and allow a non-invasive diagnosis of CRC by improving the patient’s quality of life.

Furthermore, ROS and endogenous antioxidants can be exploited as targets for the development of targeted therapies. Treatment with Hydroxy-7-methoxyflavone (HMF) induced apoptosis in HCT116 human colorectal carcinoma cells by decreasing the expression of antioxidant enzyme and upregulating the generation of mitochondrial and cytosolic ROS. The effect of HMF was reversed by pre-treatment with N-acetyl-1-cysteine, confirming the importance of ROS modulation in cancer therapy [25].

Another example of modulation of endogenous antioxidant enzymes and H_2_O_2_ production was reported by Pool et al., 2018. These authors analyzed the efficacy of Genistein, the major soy isoflavone, incorporated into PEGylated silica nanoparticles on HT29 human colon cancer cells. This formulation modulated ROS concentration by upregulating SOD and catalase activity, and simultaneously activating both apoptotic and autophagic cell death. Furthermore, nanoencapsulation improved the antioxidant activity of the biocompound, thanks to its protection and increased water solubility in the culture medium [26].

Another study also indicated that GSH inhibitors, such as buthionine sulfoximine, improve the chemosensitivity in cancer cells [27].

## 3. Exogenous Antioxidants Related to CRC Prevention and Treatment

Exogenous antioxidants can be synthetic and of natural origin.

The main natural exogenous antioxidants are contained in fruit and vegetables and are introduced with food in the diet. The role of carotenoids, vitamins and flavonoids in the prevention of CRC will be described below. Natural antioxidants are able to remove free radical intermediates and modulate enzymatic activity by suppressing early and late carcinogenesis.

Therefore, understanding the mechanism of action of these antioxidants is crucial for the development and application of new compounds in chemopreventive treatment and anticancer therapy (Table 1).

Carotenoids (carotene, lycopene, lutein, zeaxanthin) are soluble lipids; they exist as alpha and beta isoforms and are responsible for the yellow, red and orange color of fruits and vegetables. They eliminate singlet oxygen (^1^O_2_) and peroxyl radicals (ROO) [40]. There are three stages in the radical scavenger process:

- electron transfer: CAR+ROO=CAR^+^+ROO^−^
- hydrogen abstraction: CAR+ROO^−^=CAR+ROOH
- adduction: CAR+ROO=ROOCAR.

The conjugated double bonds make these compounds able to accept electrons from ROS and thus neutralize free radicals [41]. Current findings support the potential role of carotenoid in prevention and treatment of CRC [42,43].

Once digested, beta-carotene, is transformed into retinol (also known as vitamin A). Retinol inhibited tumor cell invasion and their ability to migrate through the extracellular matrix [28]. Furthermore, animal studies have shown that β-cryptoxanthin, a natural carotenoid pigment and lycopene exert the prevention of CRC through antioxidant activity and through the modulating effect on the NF-kB and SIRT1 pathways [29].

Vitamin C, known as ascorbic acid, is a water-soluble vitamin found in fresh vegetables and fruit. It reacts directly with superoxide ion O_2_ and singlet oxygen through dehydrogenation, and plays an effective role in cancer treatment and prevention. Current research is focused on identifying the mechanisms by which vitamin C is able to influence the progression of CRC. A recent study on CRC patients showed that ascorbate levels are lower in the colon tumor core than in the peripheral regions. After intravenous infusion of ascorbate, its levels increased in all tumor regions, suggesting an increase in plasma availability. Additionally, increased ascorbate levels modulated HIF activation. Since no significant adverse effects and changes in quality of life were found, these data encourage the use of vitamin C infusion rather than oral administration in complementary medicine [30].

Fat soluble Vitamin E, also known as tocopherol, exists in the, β, γ and δ forms, and is contained in food such as vegetable oil in soy, nuts and corn. It has antioxidant activity on the cell membrane; in particular, it protects cells from lipid peroxidation by stabilizing cell membranes and playing a protective role against ROS-mediated carcinogenesis [44]. Vitamin E is effective as a prevention of CRC, by reducing chronic inflammation in inflammatory bowel disease [45].

Flavonoids have a skeleton of 15-carbon, containing two benzene rings linked by a connecting chain of 3-carbon atoms (C6-C3-C6 compounds). Based on the chemical structure, degree of oxidation, and unsaturation of the connecting chain they can be classified into isoflavonoids, flavanones, flavanols, flavonols, flavones and anthocyanidins (Table 2) [46].

Flavonoids are able to chelate redox-active metals, such as copper and iron, inhibiting lipid peroxidation by scavenging peroxyl radicals (^.^ROO). They have a dual effect on ROS homeostasis, in physiological conditions they are antioxidants preventing carcinogenesis, but in oncological disease they are pro-oxidants favoring the activation of the apoptotic pathway [47,48]. Both antioxidant and pro-oxidant activities are involved in the anticancer effects of flavonoids.

Antioxidant activity can be direct or indirect. The direct antioxidant activity of flavonoids is due to the presence of phenolic hydroxyl groups that stabilize free radicals by expelling and chelating metal ions. The human body has a high level of free metal ions. 

The most abundant is iron which exists in the free form and in the form bound to the divalent (Fe (II)) or trivalent (Fe (III)) state. However, when it is available in free form, it participates in the Fenton’s reaction by becoming a source of free radicals that cause many diseases, such as colon cancer.

The same reaction is observed for Cu (II) in the free state. Flavonoids, thanks to the presence of oxygen in the ketone form and the presence of phenolic groups, are able to chelate metal ions. After chelation, flavonoids block the generation of free radicals and reduce oxidative stress and, consequently, decrease the risk of cancer development [49]. 

The indirect antioxidant activity of flavonoid is due to the inhibition of pro-oxidant enzymes such as NADPH oxidase, lipoxygenase and xanthine oxidase and to the stimulation of production or activation of antioxidant enzymes such as SOD, CAT, GR and GPx.

Some examples of flavonoids able to exert a chemopreventive effect thanks to their antioxidant activity will now be listed.

Some authors have studied the antioxidant effect of epigallocatechin-3-gallate (EGCG), contained in green tea (*Camelia sinensis*). It modulates antioxidant enzymes, such as SOD and CAT, inhibiting tumor evolution [31]. Additionally, colon cancer cells are known to have excessive iron content, which leads to cancer progression. A recent study shows that EGCG possesses an iron chelator activity in CRC and this action can be exploited for the treatment of CRC [32].

Hydroxytyrosol, a phenolic compound abundant in olive oil, enhances cellular antioxidant defenses, protecting cells from oxidative stress. Scientific evidence on Caco-2 cells has shown that hydroxytyrosol is able to counteract the ROS-mediated cytotoxicity of food acrylamide [33].

The flavonol quercetin, abundant in onions, apples, tomatoes, broccoli and citrus fruits, has been shown to have a chemopreventive effect against cancer [50]. There are many mechanisms underlying the chemopreventive effect of quercetin: for example, quercetin inhibits hepatocarcinogenesis by up-regulating the enzymatic and not-enzymatic antioxidant defense system [34]. The chemopreventive effects of quercetin in colorectal cancer are due to modulation of signaling cascades, gene expressions of cell proliferation, differentiation, apoptosis and suppression of chronic inflammation, metastasis, and angiogenesis [51].

Furthermore, the catechins and procyanidins of Cocoa protected Caco-2 cells [35]. A study conducted on cellular and animal models and on human cohorts has shown that cocoa and flavonols derived from cocoa, modify the inflammatory process. They are useful compounds for controlling the development and progression of tumor-associated inflammation, such as CRC [36].

Cyanidin and delphinidin inhibited glutathione reductase and depleted glutathione of drug sensitive and multi-resistant metastatic colorectal cancer cells (LoVo and LoVo ADR). However, they were not toxic to the cells of a primary tumor site, such as Caco-2 cells. This study showed that the dual antioxidant/pro-oxidant effect of anthocyanidins was cell-type specific. Anthocyanidins had antioxidant activity against Caco-2 cells, characterized by a low proliferation rate and low malignancy, protecting them from oxidative stress. However, they had a pro-oxidant action against malignant and drug resistant cells with active metabolism [52].

Other compounds such as sesamol, curcumin, resveratrol, have a dose-dependent paradoxical effect. Sesamol, a phenolic compound contained in sesame seeds, had a pro-oxidant effect on human colorectal carcinoma HTC116 cells at high concentrations (0.5–10 mM); induction of mitochondrial apoptosis was due to the intracellular O_2_^−^ generation. Even a low concentration of sesamol had an antioxidant effect due to the presence of hydroxyl group along with a methylene dioxy group in its structure [37].

Curcumin, the main biologically active compound of *Curcuma longa*, has an anti-radical effect thanks to the CH_2_ group of the beta-diketone fraction [53]. Its antioxidant activity makes it a promising candidate for colon diseases prevention and combination therapy [38,54]. Unfortunately, its poor bioavailability, due to metabolism and its poor adsorption, limits its use and favors the synthesis of news derivatives [55].

Finally, resveratrol, a polyphenol contained in grapes and red wine, inhibits the formation of superoxide anion and hydrogen peroxide in the body. Furthermore, it reduces lipid peroxidation, decreasing inflammation of the intestinal mucous and preventing the development of colon neoplasia [39,56]. Several resveratrol analogues have been synthesized by adding functional groups to improve chemopreventive efficacy [57].

## 4. Experimental Evidence of Dual Antioxidant or Pro-Oxidant Effect of *Prunus spinosa* Extract on Colorectal Cancer Cells 

In the following paragraph, we report an example of a natural compound that has been shown in our previous studies to have an antiproliferative and antitumor effect both in vitro and in vivo models. In particular, we will investigate the antioxidant effect of *Prunus spinosa* Trigno ecotype extract on the human colorectal cell line, HCT116.

**Figure 4:**
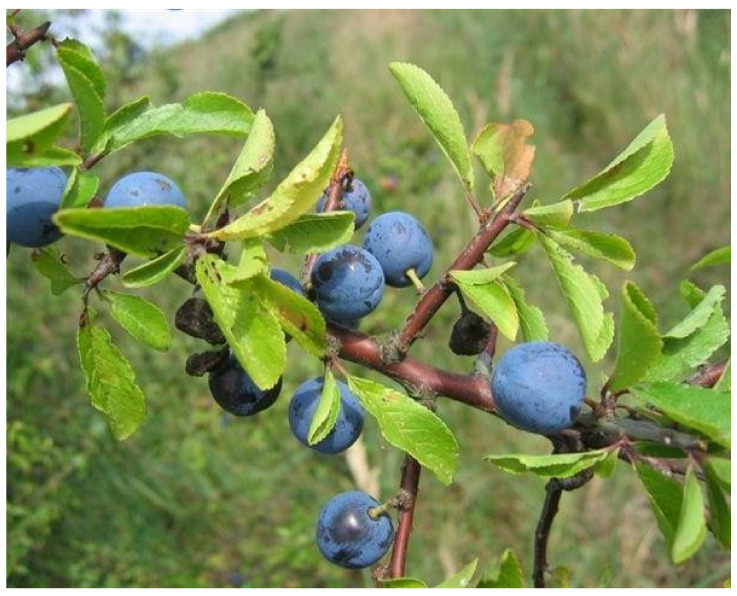
*Prunus spinosa* Trigno ecotype (PsT) plant, from Bagnoli del Trigno, Molise, Italy.

*Prunus spinosa* belongs to the *Rosaceae* family, a very widespread plant in Italy, but also present in others European countries and in the temperate regions of Asia, where it is used in the treatment of hypertension and gastrointestinal disorders (Figure 4) [58]. The active compounds of its extract mainly consist of phenolic acids, flavonoids, and anthocyanins [59].

Recent literature data showed that methanolic extract of *Prunus spinosa* flowers was effective against glioblastoma cells by inducing an antioxidant effect [60]. 

Another hepatocarcinoma cell study showed that *Prunus spinosa* blackthorn flower extract had pro-oxidant behavior within the applied concentration range and induced apoptotic and necrotic cell death [61].

Furthermore, the low molecular weight polyphenols contained in the *Prunus spinosa* extract had an antioxidant and protective effect against fibrinogen and other human plasma components [62].

To improve biocompatibility and increase tissue accumulation, *Prunus spinosa* extract has also been loaded into biomimetic nanoparticles. These formulations have been shown to have a greater anti-inflammatory, antioxidant activities and a more wound-healing effect compared to free formulation [63].

The antioxidant properties and antimicrobial activity of some components such as the anthocyanins of the epicarp of the *Prunus spinosa* fruit allow this extract to be used as color in industrial food production [64,65].

In our experimental model we used the hydroalcoholic extract of drupes of *Prunus spinosa* Trigno ecotype (PsT) from Bagnoli del Trigno, Molise, Italy. Liquid chromatography coupled to mass spectrometry analysis identified the chemical composition of PsT. The plant extract is characterized by the presence of active compounds as phenolic acids, flavonoids and anthocyanins. In particular, there are greater quantities of flavone/ols compounds (64.62 ± 0.58 mg/100 g of dry weight), phenolic acids compounds (38.36 ± 0.19 mg/100 g), and anthocyanins group (0.63 μg/100 g) [59].

Our previous study showed that *Prunus spinosa* drupe extract combined with a nutraceutical activator complex (NAC) consisting of amino acids, vitamins and mineral salts, was effective against various cancer cell lines, such as colorectal, uterine cervix, and bronchoalveolar cells. The MTT and clonogenic assay showed a reduction in cell viability, especially when the *Prunus spinosa* extract was administered with NAC vehicle. Results of Annexin V assay and cell cycle analysis on two colon carcinoma cell lines demonstrated induction of apoptosis. Furthermore, cell survival tests showed that this complex was not toxic on non-cancerous human cell lines [59].

The anticancer effect of *Prunus spinosa* extract in combination with NAC has been extensively studied on 2D, 3D and in vivo models of colon cancer [6]. The treatments on the 2D model confirmed the previous results and showed the inhibiting effect on the migration ability of the HCT116 cell. *Prunus spinosa* extract plus NAC altered the continuity of the outer cell membrane of the HCT116 spheroids, inducing cell death by apoptosis. Furthermore, treatment of immunodeficient mice carrying colon rectal cancer reduced tumor growth. These results suggested that this nutraceutical complex could be used in clinical protocols in association with chemotherapy for the treatment of colon cancer. To this end, the concentration of *Prunus spinosa* extract 10 mg/mL (PsT 10) plus NAC has been patented as Trigno M [66].

**Figure 5:**
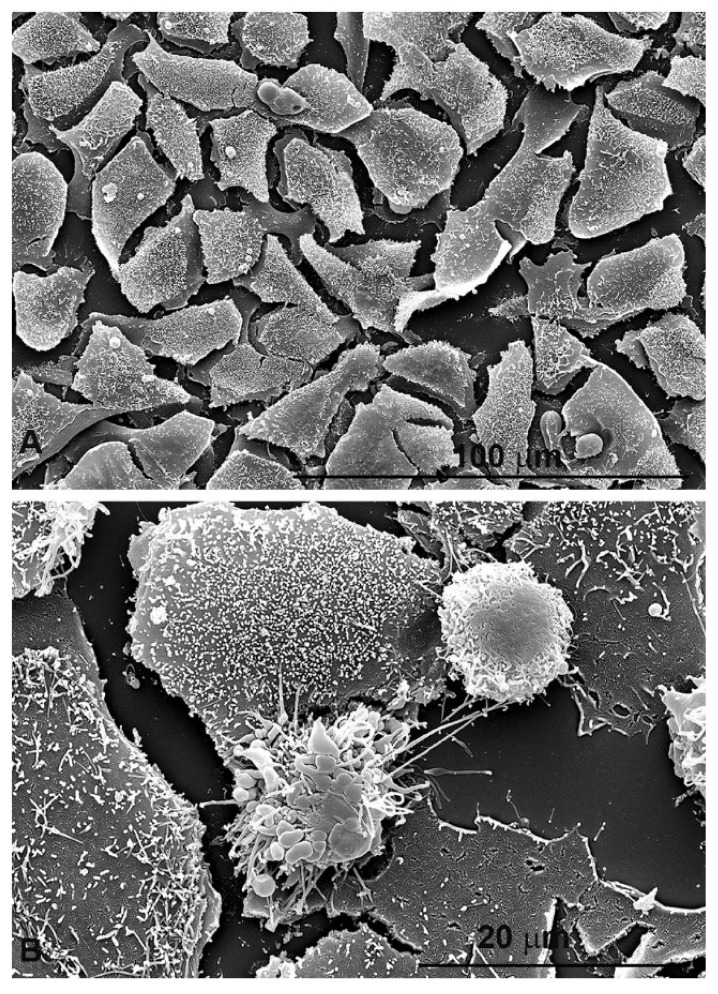
Scanning electron microscopic observations of morphological changes induced by Prunus sp. extract on HCT116 cells. (**A**) untreated HCT116 cells; (**B**) HCT116 cells treated with PsT 10 mg/mL plus NAC for 24 h. Cells were fixed with glutaraldehyde and sucrose in cacodylate buffer. After post-fixation with O_s_O_4_ for 30 min, cells were dehydrated through graded ethanol concentration, critical point-dried in CO_2_ (CPD 030 Balzers device, Bal-Tec, Balzers), and gold coated by sputtering (SCD040 Balzers device, Bal-Tec). The observations were performed with a field emission gun scanning electron microscope (Quanta 200 Inspect, FEI Company, Eindhoven, The Netherlands). For detailed description see Condello et al., 2015 [67].

Before proceeding with the study on the role of oxidative stress in colon cancer 2D line, we verified the effect on cell morphology induced by PsT 10 plus NAC treatment by scanning electron microscopy (SEM). HCT116 cells treated with PsT 10 plus NAC for 24 h (Figure 5B) were morphologically modified compared to control cells (Figure 5A). This image clearly indicated severe signs of cellular distress and apoptosis.

Now, to clarify the mechanisms related to cell death due to apoptosis quantified after treatment with PsT 10 plus NAC, we have undertaken the study of oxidative stress using the flow cytometry technique.

We performed flow cytometric analyses of superoxide content by dihydrorhodamine 123 (DHR123) probe, the loss of mitochondrial membrane potential by tetramethylrhodamine methyl ester (TMRM) cation probe, and evaluated GSH content by monochlorobimane (MCB) probe.

**Figure 6:**
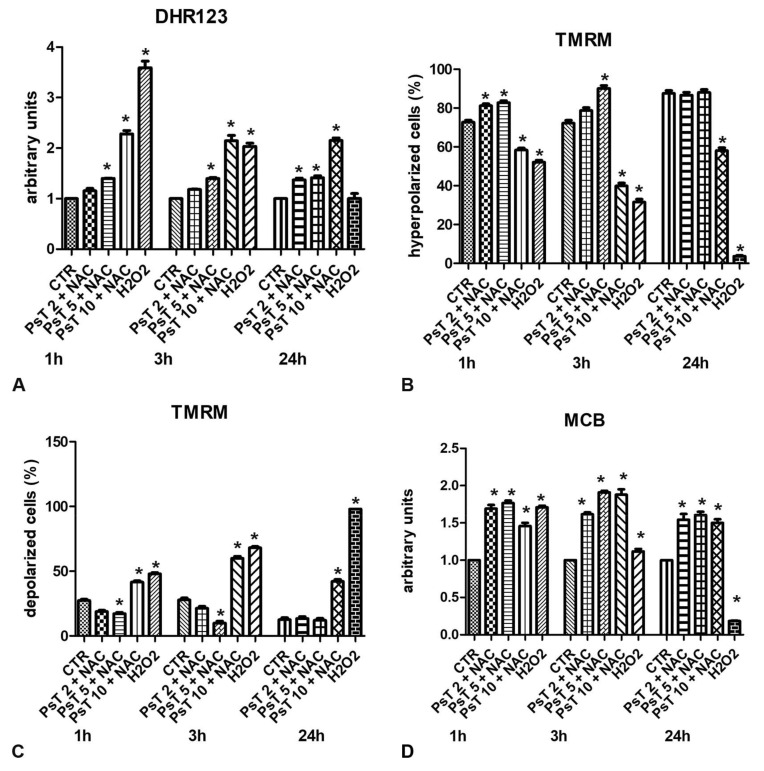
Flow cytometric analysis of oxidative stress after HCT116 cells treatment with increased concentration of *Prunus spinosa* extract plus nutraceutical activator complex (NAC) (PsT 2 mg/mL + NAC; PsT 5 mg/mL + NAC; PsT 10 mg/mL + NAC) for 1, 3 and 24h. (**A**) Superoxide content by dihydrorhodamine 123 (DHR123) probe; (**B**) hyperpolarized cell percentage by tetramethylrhodamine methyl ester (TMRM) cation probe; (**C**) depolarized cell percentage by tetramethylrhodamine methyl ester (TMRM) cation probe; (**D**) GSH content by monochlorobimane (MCB) probe. After treatment, cells were incubated with DHR123, or TMRM or MCB and then analyzed by a BDLSRII flow cytometer (Becton, Dickinson & Company, Franklin Lakes, NJ, USA). For detailed materials and methods see De Berardis et al., 2010 [73]. The results obtained from three independent experiments were expressed as mean±standard deviation. One-way Analysis of Variance (ANOVA), and Dunnett post hoc analysis are applied to reveal differences between all treated and control samples, using GraphPad Prism 5 software (GraphPad, San Diego, CA, USA). The alpha level was set at *p* < 0.05. *: *p* < 0.05 *vs* control.

As shown in Figure 6A, the increase of DHR123 content is dose-dependent in all times of treatment. The highest signal was obtained after treatment with PsT 10 mg/mL plus NAC for 1 h.

Using the TMRM probe, we observed that treatments with PsT 2 mg/mL plus NAC or PsT 5 mg/mL plus NAC for 1, 3 and 24 h induced hyperpolarization of the mitochondrial membrane potential, in agreement with the weak increase in ROS determination (Figure 6B). After treatment with PsT 10 mg/mL plus NAC for 24 h, the mitochondrial membrane potential was depolarized (Figure 6C). The mitochondrial depolarization observed after treatment with PsT 10 plus NAC agree the previously demonstrated induction of apoptosis. Several other authors demonstrated a direct correlation between the alteration of mitochondrial function and the induction of cell death in the disability of aging and degenerative diseases [68,69].

The assessment of the reduced glutathione (GSH) content is very important because it protects cells from oxidative damage and maintains a number of vital functions. Therefore, as regards the evaluation of the GSH content by MCB probe, its value increased after each concentration and in all treatment times (Figure 6D). As shown in the figure, GSH levels after treatment with PsT at a concentration (10 mg/mL plus NAC) were lower than at low concentrations (PsT 2 mg/mL + NAC, PsT 3 mg/mL + NAC).

Based on these results, we have shown that the nutraceutical complex based on *Prunus spinosa*, at low concentrations, has a weak antioxidant effect which tends to reduce with increasing concentration. The pro-oxidant effect was observed and quantified at 10 mg/mL concentration, when antitumor activity was revealed.

The experimental part we carried out on the nutraceutical compound based on *Prunus spinosa*, rich in various substances with high antioxidant power and also in kaempferol and quercetin, shows the importance of a careful evaluation of the concentrations to be used both as a supplement and as a support in clinical trials [6]. The same compound, in fact, can modify its behavior according to the cellular physiological situation.

An important question to address, and this also applies to *Prunus spinosa*, is how natural polyphenolic compounds transform when they come into contact with the human gut microbiota [70]. Many works, in fact, demonstrate the importance of the intestinal microbiota in subjects who follow a diet rich in polyphenols and fatty acids in dealing with diseases such as cardiovascular, neurodegenerative and colon cancer. The biotransformation of polyphenols by the microbiota depends in fact on the type of bacteria present and on how the intestinal flora is able to metabolize the polyphenols in order to obtain a beneficial effect on the metabolism of lipids and carbohydrates. Indeed, tumors present an alteration of these mechanisms [71]. One of the most studied compounds is quercetin which when bio accessible to the colon microbiota is metabolized and becomes commensal for various intestinal bacteria [72]. Certainly in-depth studies and a better understanding of the interaction between polyphenols, metabolites and intestinal microbiota will be very useful in the prevention and treatment of colon cancer.

## 5. Absorption Limit and In Vivo Bioavailability for Flavonoids and Natural Antioxidant Products

Flavonoids, as mentioned above, are a group of widely studied phytochemicals with many beneficial properties on human health such as anti-inflammatory, antitumor, cholesterol-lowering anti-aging. Unfortunately, however, they have a low bioavailability. In fact, it is known that phase 2 metabolism alters the bioavailability of flavonoids in humans [74]. Most flavonoids undergo sulfation, methylation, and glucuronidation reactions in the small intestine and liver [75]. Phase 2 metabolic reactions are catalyzed by enzymes such as UDP-glucuronosyltransferase/UGT and sulfotransferase/SULT which produce highly hydrophilic conjugates such as glucuronides and sulfates that facilitate the excretion of flavonoids from the human body [76].

Furthermore, transporters such as BCRP and MRP play an important role in the pharmacokinetics and bioavailability of food polyphenols [77]. To enhance the bioavailability of natural antioxidants such as polyphenols efforts are being made to improve intestinal absorption and to improve their metabolic stability, such as new delivery systems by encapsulation [78].

From this short dissertation we understand that it is very difficult to predict the bioavailability of these compounds over time, so it is essential to increase this factor to fully exploit their therapeutic benefits in the prevention and treatment of colon cancer.

## 6. Conclusions

In conclusion, we can state that many compounds, mentioned above, show a behavior defined as paradoxical. The paradoxical aspects of antioxidant molecules are linked to ROS mechanisms in normal and cancer cells. In some cases, in fact, either the paradoxical antioxidant role or the pro-oxidant one is responsible for the anti-tumor action. This dual behavior is triggered by the complex mechanism of antioxidant enzymes present in cell physiology, which explains how their overexpression can promote disease or their elimination can be healthy. Health and disease really do arise from such sophisticated molecular mechanisms of redox biology and metabolic homeostasis.

## Figures and Tables

**Table 1:** Summary table of the main exogenous antioxidants.

Exogenous Antioxidants	Experimental Models	Dose/Concentration	Results	Reference
β-carotene	Colorectal cancer cells (LoVo)	1–10 μM	Reduced invasiveness	Pharm et al., 2013 [28]
β-cryptoxanthin/lycopene	Animal models	-	Chemoprevention and anticancer activity	Lim et al., 2020 [29]
Ascorbate	Patients	1 g/kg	Improvement of tumor biology	Dachs et al., 2021 [30]
Epigallocatechin-3-gallate	Animal models	100 mg/kg	Decreased lipid peroxides	Lambert and Elias, 2010 [31]
Epigallocatechin-3-gallate	Colorectal cancer cells (HT-29)	88.1 μM	Iron chelation activity	Nesran et al., 2020 [32]
Hydroxytyrosol	Colon cells (Caco-2)	5–100 μM	Chemoprevention	Rodríguez-Ramiro et al., 2011 [33]
Quercetin	Animal models	2–50 mg/kg	Chemoprevention	Vásquez-Garzón et al., 2009 [34]
Cocoa	Cells, animal models, patients	-	Anti-inflammatory effect	Martín et al., 2016 [35] Goya et al., 2016 [36]
Sesamol	Colorectal cancer cells (HCT-116)	0.5–10 mM	Apoptosis	Khamphio et al., 2016 [37]
Curcumin	Patients	-	Chemosensitizing effect	Mansouri et al., 2020 [38]
Resveratrol	Animal models	7.2 mg/kg	Chemoprevention	Rytsyk et al., 2020 [39]

**Table 2:** Classification of flavonoids.

Isoflavonoids	Flavanones	Flavanols	Flavonol	Flavone	Anthocyanidins
Genistein	Hesperetin	Cathecin	Quercetin	Apigenin	Cyanidin
Daidzein	Naringenin	Epicatechin	Kaempfrol	Luteolin	Malvidin
			Galangin	Chrysin	Delphinidin
			Fisetin		Petunidin
			Myricetin		Peonidin

## Data Availability

Not applicable.

## References

[1] Sawicki T., Ruszkowska M., Danielewicz A., Niedźwiedzka E., Arłukowicz T., Przybyłowicz K. (2021). A Review of Colorectal Cancer in Terms of Epidemiology, Risk Factors, Development, Symptoms and Diagnosis. Cancers.

[2] Testa U., Pelosi E., Castelli G. (2018). Colorectal Cancer: Genetic Abnormalities, Tumor Progression, Tumor Heterogeneity, Clonal Evolution and Tumor-Initiating Cells. Med. Sci..

[3] Wang Y., Qi H., Liu Y., Duan C., Liu X., Xia T., Chen D., Piao H.-L., Liu H.-X. (2021). The double-edged roles of ROS in cancer prevention and therapy. Theranostics.

[4] Basak D., Uddin M.N., Hancock J. (2020). The Role of Oxidative Stress and Its Counteractive Utility in Colorectal Cancer (CRC). Cancers.

[5] Ganesan K., Jayachandran M., Xu B. (2020). Diet-Derived Phytochemicals Targeting Colon Cancer Stem Cells and Microbiota in Colorectal Cancer. Int. J. Mol. Sci..

[6] Condello M., Pellegrini E., Spugnini E.P., Baldi A., Amadio B., Vincenzi B., Occhionero G., Delfine S., Mastrodonato F., Meschini S. (2019). Anticancer activity of “Trigno M”, extract of Prunus spinosa drupes, against in vitro 3D and in vivo colon cancer models. Biomed. Pharmacother..

[7] Marengo B., Nitti M., Furfaro A.L., Colla R., De Ciucis C., Marinari U.M., Pronzato M.A., Traverso N., Domenicotti C. (2016). Redox Homeostasis and Cellular Antioxidant Systems: Crucial Players in Cancer Growth and Therapy. Oxid. Med. Cell. Longev..

[8] Lubos E., Loscalzo J., Handy D.E. (2011). Glutathione Peroxidase-1 in Health and Disease: From Molecular Mechanisms to Therapeutic Opportunities. Antioxidants Redox Signal..

[9] Warsinggih, Irawan B., Labeda I., Lusikooy R.E., Sampetoding S., Kusuma M.I., Uwuratuw J.A., Syarifuddin E., Prihantono, Faruk M. (2020). Association of superoxide dismutase enzyme with staging and grade of differentiation colorectal cancer: A cross-sectional study. Ann. Med. Surg..

[10] Jaramillo M.C., Zhang D.D. (2013). The emerging role of the Nrf2–Keap1 signaling pathway in cancer. Genes Dev..

[11] Kerins M.J., Ooi A. (2018). A catalogue of somatic NRF2 gain-of-function mutations in cancer. Sci. Rep..

[12] Gamcsik M.P., Kasibhatla M.S., Teeter S., Colvin O.M. (2012). Glutathione levels in human tumors. Biomarkers.

[13] Xiang Z., Kang Q., Xiang X. (2015). Gene and protein expression in the oxaliplatin-resistant HT29/L-OHP human colon cancer cell line. Genet. Mol. Res..

[14] Gazzin S., Vitek L., Watchko J., Shapiro S.M., Tiribelli C. (2016). A Novel Perspective on the Biology of Bilirubin in Health and Disease. Trends Mol. Med..

[15] Wagner K.-H., Wallner M., Moelzer C., Gazzin S., Bulmer A.C., Tiribelli C., Vitek L. (2015). Looking to the horizon: The role of bilirubin in the development and prevention of age-related chronic diseases. Clin. Sci..

[16] Khoei N.S., Anton G., Peters A., Freisling H., Wagner K.-H. (2020). The Association between Serum Bilirubin Levels and Colorectal Cancer Risk: Results from the Prospective Cooperative Health Research in the Region of Augsburg (KORA) Study in Germany. Antioxidants.

[17] Ollinger R., Kogler P., Troppmair J., Hermann M., Wurm M., Drasche A., Konigsrainer I., Amberger A., Weiss H., Ofner D. (2007). Bilirubin Inhibits Tumor Cell Growth via Activation of ERK. Cell Cycle.

[18] Keshavan P., Schwemberger S.J., Smith D.L., Babcock G.F., Zucker S.D. (2004). Unconjugated bilirubin induces apoptosis in colon cancer cells by triggering mitochondrial depolarization. Int. J. Cancer.

[19] Grant D.J., Bell D.A. (2000). Bilirubin UDP-Glucuronosyltransferase 1A1 Gene Polymorphisms: Susceptibility to Oxidative Damage and Cancer?. Mol Carcinog..

[20] Jiang M., Ren L., Chen S., Li G. (2021). Serum Uric Acid Levels and Risk of Eight Site-Specific Cancers: A Mendelian Randomization Study. Front. Genet..

[21] Üstüner M.A., Dogan L. (2020). Relationship of Preoperative Serum Uric Acid Level with Survival in Colorectal Cancer. J. Coll. Physicians Surg. Pak..

[22] Paronetto M.P., Dimauro I., Grazioli E., Palombo R., Guidotti F., Fantini C., Sgrò P., De Francesco D., Di Luigi L., Capranica L. (2020). Exercise-mediated downregulation of MALAT1 expression and implications in primary and secondary cancer prevention. Free. Radic. Biol. Med..

[23] Gutschner T., Hämmerle M., Diederichs S. (2013). MALAT1 — a paradigm for long noncoding RNA function in cancer. J. Mol. Med..

[24] Zińczuk J., Maciejczyk M., Zaręba K., Romaniuk W., Markowski A.R., Kędra B., Zalewska A., Pryczynicz A., Matowicka-Karna J., Guzińska-Ustymowicz K. (2019). Antioxidant Barrier, Redox Status, and Oxidative Damage to Biomolecules in Patients with Colorectal Cancer. Can Malondialdehyde and Catalase Be Markers of Colorectal Cancer Advancement?. Biomolecules.

[25] Bhardwaj M., Kim N.-H., Paul S., Jakhar R., Han J., Kang S.C. (2016). 5-Hydroxy-7-Methoxyflavone Triggers Mitochondrial-Associated Cell Death via Reactive Oxygen Species Signaling in Human Colon Carcinoma Cells. PLoS ONE.

[26] Pool H., Campos-Vega R., Herrera-Hernández M.G., García-Solis P., García-Gasca T., Sánchez I.C., Lu-na-Bárcenas G., Vergara-Castañeda H. (2018). Development of Genistein-PEGylated Silica Hybrid Nanomaterials with Enhanced Antioxidant and Antiproliferative Properties on HT29 Human Colon Cancer Cells. Am. J. Transl. Res..

[27] Narayanankutty A., Job J.T., Narayanankutty V. (2019). Glutathione, an Antioxidant Tripeptide: Dual Roles in Carcinogenesis and Chemoprevention. Curr. Protein Pept. Sci..

[28] Pham D.N.T., Leclerc D., Lévesque N., Deng L., Rozen R. (2013). β,β-Carotene 15,15′-monooxygenase and its substrate β-carotene modulate migration and invasion in colorectal carcinoma cells. Am. J. Clin. Nutr..

[29] Lim J.Y., Wang X.-D. (2020). Mechanistic understanding of β-cryptoxanthin and lycopene in cancer prevention in animal models. Biochim. Biophys. Acta (BBA) Mol. Cell Biol. Lipids.

[30] Dachs G.U., Gandhi J., Wohlrab C., Carr A.C., Morrin H.R., Pullar J.M., Bayer S.B., Eglinton T.W., Robinson B.A., Vissers M.C.M. (2021). Vitamin C Administration by Intravenous Infusion Increases Tumor Ascorbate Content in Patients with Colon Cancer: A Clinical Intervention Study. Front. Oncol..

[31] Lambert J.D., Elias R.J. (2010). The antioxidant and pro-oxidant activities of green tea polyphenols: A role in cancer prevention. Arch. Biochem. Biophys..

[32] Nesran Z.N.M., Shafie N.H., Tohid S.F.M., Norhaizan M.E., Ismail A. (2020). Iron Chelation Properties of Green Tea Epigallocatechin-3-Gallate (EGCG) in Colorectal Cancer Cells: Analysis on Tfr/Fth Regulations and Molecular Docking. Evid. Based Complementary Altern. Med..

[33] Rodríguez-Ramiro I., Martín M.A., Ramos S., Bravo L., Goya L. (2011). Olive oil hydroxytyrosol reduces toxicity evoked by acrylamide in human Caco-2 cells by preventing oxidative stress. Toxicology.

[34] Vásquez-Garzón V.R., Arellanes-Robledo J., García-Román R., Aparicio-Rautista D.I., Villa-Treviño S. (2009). Inhibition of reactive oxygen species and pre-neoplastic lesions by quercetin through an antioxidant defense mechanism. Free Radic. Res..

[35] Martín M., Ángeles, Goya L., Ramos S. (2016). Preventive Effects of Cocoa and Cocoa Antioxidants in Colon Cancer. Diseases.

[36] Goya L., Martín M., Ángeles, Sarriá B., Ramos S., Mateos R., Bravo L. (2016). Effect of Cocoa and Its Flavonoids on Biomarkers of Inflammation: Studies of Cell Culture, Animals and Humans. Nutrients.

[37] Khamphio M., Barusrux S., Weerapreeyakul N. (2016). Sesamol induces mitochondrial apoptosis pathway in HCT116 human colon cancer cells via pro-oxidant effect. Life Sci..

[38] Mansouri K., Rasoulpoor S., Daneshkhah A., Abolfathi S., Salari N., Mohammadi M., Shabani S. (2020). Clinical effects of curcumin in enhancing cancer therapy: A systematic review. BMC Cancer.

[39] Rytsyk O., Soroka Y., Shepet I., Vivchar Z., Andriichuk I., Lykhatskyi P., Fira L., Nebesna Z., Kramar S., Lisnychuk N. (2020). Experimental Evaluation of the Effectiveness of Resveratrol as an Antioxidant in Colon Cancer Prevention. Nat. Prod. Commun..

[40] Milani A., Basirnejad M., Shahbazi S., Bolhassani A. (2016). Carotenoids: Biochemistry, pharmacology and treatment. Br. J. Pharmacol..

[41] Rutz J.K., Borges C.D., Zambiazi R.C., Da Rosa C.G., Da Silva M.M. (2016). Elaboration of microparticles of carotenoids from natural and synthetic sources for applications in food. Food Chem..

[42] Kim D., Kim Y., Kim Y. (2019). Effects of β-carotene on Expression of Selected MicroRNAs, Histone Acetylation, and DNA Methylation in Colon Cancer Stem Cells. J. Cancer Prev..

[43] Saini R.K., Keum Y.-S., Daglia M., Rengasamy K.R. (2020). Dietary carotenoids in cancer chemoprevention and chemotherapy: A review of emerging evidence. Pharmacol. Res..

[44] Espley R.V., Butts C.A., Laing W.A., Martell S., Smith H., McGhie T.K., Zhang J., Paturi G., Hedderley D., Bovy A. (2014). Dietary Flavonoids from Modified Apple Reduce Inflammation Markers and Modulate Gut Microbiota in Mice. J. Nutr..

[45] Yang C.S., Luo P., Zeng Z., Wang H., Malafa M., Suh N. (2020). Vitamin E and cancer prevention: Studies with different forms of tocopherols and tocotrienols. Mol. Carcinog..

[46] Kopustinskiene D.M., Jakstas V., Savickas A., Bernatoniene J. (2020). Flavonoids as anticancer agents. Nutrients.

[47] Hadi S.F.A.S.M., Asad S.F., Singh S., Ahmad A. (2000). Putative Mechanism for Anticancer and Apoptosis-Inducing Properties of Plant-Derived Polyphenolic Compounds. IUBMB Life.

[48] Link A., Balaguer F., Goel A. (2010). Cancer chemoprevention by dietary polyphenols: Promising role for epigenetics. Biochem. Pharmacol..

[49] Selvaraj S., Krishnaswamy S., Devashya V., Sethuraman S., Krishnan U.M. (2013). Flavonoid-Metal Ion Complexes: A Novel Class of Therapeutic Agents. Med. Res. Rev..

[50] Rather R.A., Bhagat M. (2020). Quercetin as an innovative therapeutic tool for cancer chemoprevention: Molecular mechanisms and implications in human health. Cancer Med..

[51] Darband S.G., Kaviani M., Yousefi B., Sadighparvar S., Pakdel F.G., Attari J.A., Mohebbi I., Naderi S., Majidinia M. (2018). Quercetin: A functional dietary flavonoid with potential chemo-preventive properties in colorectal cancer. J. Cell. Physiol..

[52] Cvorovic J., Tramer F., Granzotto M., Candussio L., Decorti G., Passamonti S. (2010). Oxidative stress-based cytotoxicity of delphinidin and cyanidin in colon cancer cells. Arch. Biochem. Biophys..

[53] López-Lázaro M. (2008). Anticancer and carcinogenic properties of curcumin: Considerations for its clinical development as a cancer chemopreventive and chemotherapeutic agent. Mol. Nutr. Food Res..

[54] Jakubczyk K., Drużga A., Katarzyna J., Skonieczna-Żydecka K. (2020). Antioxidant Potential of Curcumin—A Meta-Analysis of Randomized Clinical Trials. Antioxidants.

[55] Kelkel M., Jacob C., Dicato M., Diederich M. (2010). Potential of the Dietary Antioxidants Resveratrol and Curcumin in Prevention and Treatment of Hematologic Malignancies. Molecules.

[56] Ko J.-H., Sethi G., Um J.-Y., Shanmugam M.K., Arfuso F., Kumar A.P., Bishayee A., Ahn K.S. (2017). The Role of Resveratrol in Cancer Therapy. Int. J. Mol. Sci..

[57] Fulda S. (2010). Resveratrol and derivatives for the prevention and treatment of cancer. Drug Discov. Today.

[58] Calvo M., Cavero R.Y. (2014). Medicinal plants used for cardiovascular diseases in Navarra and their validation from Official sources. J. Ethnopharmacol..

[59] Meschini S., Pellegrini E., Condello M., Occhionero G., Delfine S., Condello G., Mastrodonato F. (2017). Cytotoxic and Apoptotic Activities of Prunus spinosa Trigno Ecotype Extract on Human Cancer Cells. Molecules.

[60] Karakas N., Okur M.E., Ozturk I., Ayla S., Karadağ A.E., Çiçek Polat D. (2019). Antioxidant activity and cytotoxic effects of Prunus spinosa L. fruit extract on various cancer cell lines. Medeni. Med J..

[61] Murati T., Miletić M., Kolarić J., Lovrić V., Kovačević D.B., Putnik P., Jurčević I.L., Đikić D., Dragović-Uzelac V., Kmetič I. (2019). Toxic activity of Prunus spinosa L. flower extract in hepatocarcinoma cells. Arch. Ind. Hyg. Toxicol..

[62] Marchelak A., Owczarek A., Matczak M., Pawlak A., Kolodziejczyk-Czepas J., Nowak P., Olszewska M.A. (2017). Bioactivity Potential of Prunus spinosa L. Flower Extracts: Phytochemical Profiling, Cellular Safety, Pro-inflammatory Enzymes Inhibition and Protective Effects Against Oxidative Stress In Vitro. Front. Pharmacol..

[63] Tiboni M., Coppari S., Casettari L., Guescini M., Colomba M., Fraternale D., Gorassini A., Verardo G., Ramakrishna S., Guidi L. (2020). *Prunus spinosa* Extract Loaded in Biomimetic Nanoparticles Evokes In Vitro Anti-Inflammatory and Wound Healing Activities. Nanomaterials.

[64] Backes E., Leichtweis M.G., Pereira C., Carocho M., Barreira J.C., Kamal Genena A., José Baraldi I., Filomena Barreiro M., Barros L., Ferreira I.C. (2020). Ficus carica L. and Prunus spinosa L. extracts as new anthocyanin-based food colorants: A thorough study in confectionery products. Food Chem..

[65] Pozzo L., Russo R., Frassinetti S., Vizzarri F., Árvay J., Vornoli A., Casamassima D., Palazzo M., DELLA Croce C.M., Longo V. (2019). Wild Italian Prunus spinosa L. Fruit Exerts In Vitro Antimicrobial Activity and Protects Against In Vitro and In Vivo Oxidative Stress. Foods.

[66] Meschini S., Mastrodonato F. (2015). Estratti di Prunus spinosa ad Attività Antitumorale. Italian Patent.

[67] Condello M., Multari G., Gallo F.R., Arancia G., Meschini S. (2015). High-performance thin-layer chromatography for the evaluation of voacamine intracellular concentration related to its cytotoxic effect. J. Pharm. Biomed. Anal..

[68] Zahm J.-M., Baconnais S., Monier S., Bonnet N., Bessède G., Gambert P., Puchelle E., Lizard G. (2003). Chronology of cellular alterations during 7-ketocholesterol-induced cell death on A7R5 rat smooth muscle cells: Analysis by time lapse-video microscopy and conventional fluorescence microscopy. Cytom. Part A.

[69] Huang M.L.-H., Chiang S., Kalinowski D.S., Bae D.-H., Sahni S., Richardson D.R. (2019). The Role of the Antioxidant Response in Mitochondrial Dysfunction in Degenerative Diseases: Cross-Talk between Antioxidant Defense, Autophagy, and Apoptosis. Oxidative Med. Cell. Longev..

[70] Yammine A., Namsi A., Vervandier-Fasseur D., Mackrill J., Lizard G., Latruffe N. (2021). Polyphenols of the Mediterranean Diet and Their Metabolites in the Prevention of Colorectal Cancer. Molecules.

[71] Koudoufio M., Desjardins Y., Feldman F., Spahis S., Delvin E., Levy E. (2020). Insight into Polyphenol and Gut Microbiota Crosstalk: Are Their Metabolites the Key to Understand Protective Effects Against Metabolic Disorders?. Antioxidants.

[72] Shabbir U., Rubab M., Daliri E.B.-M., Chelliah R., Javed A., Oh D.-H. (2021). Curcumin, Quercetin, Catechins and Metabolic Diseases: The Role of Gut Microbiota. Nutrients.

[73] De Berardis B., Civitelli G., Condello M., Lista P., Pozzi R., Arancia G., Meschini S. (2010). Exposure to ZnO nanoparticles induces oxidative stress and cytotoxicity in human colon carcinoma cells. Toxicol. Appl. Pharmacol..

[74] Manach C., Williamson G., Morand C., Scalbert A., Rémésy C. (2005). Bioavailability and bioefficacy of polyphenols in humans. I. Review of 97 bioavailability studies. Am. J. Clin. Nutr..

[75] Mullen W., Edwards C.A., Crozier A. (2006). Absorption, excretion and metabolite profiling of methyl-, glucuronyl-, glucosyl- and sulpho-conjugates of quercetin in human plasma and urine after ingestion of onions. Br. J. Nutr..

[76] Hu M., Wu B., Liu Z. (2017). Bioavailability of Polyphenols and Flavonoids in the Era of Precision Medicine. Mol. Pharm..

[77] Hussain S., Sulaiman A., Alhaddad H., Alhadidi Q. (2016). Natural polyphenols: Influence on membrane transporters. J. Intercult. Ethnopharmacol..

[78] Williamson G., Kay C.D., Crozier A. (2018). The Bioavailability, Transport, and Bioactivity of Dietary Flavonoids: A Review from a Historical Perspective. Compr. Rev. Food Sci. Food Saf..

